# Insights into the Saliva of the Brown Marmorated Stink Bug *Halyomorpha halys* (Hemiptera: Pentatomidae)

**DOI:** 10.1371/journal.pone.0088483

**Published:** 2014-02-26

**Authors:** Michelle Peiffer, Gary W. Felton

**Affiliations:** Department of Entomology, Penn State University, University Park, Pennsylvania, United States of America; AgroParisTech, France

## Abstract

We examined the salivary gland structure of the brown marmorated stink bug (Pentatomidae: *Halyomorpha halys*) and developed methods for independent collection of watery saliva and sheath saliva. This stink bug has become a serious invasive pest of agriculture in the United States and its saliva is largely responsible for the damage it causes. We determined by protein gel analysis and shotgun proteomics that the suite of proteins comprising the sheath and watery saliva are very distinct. Our results indicate that a substantial amount of sheath proteins are derived from tomato when stink bugs feed on tomato fruit. Consequently, the sheath saliva is comprised of both insect and plant-derived proteins. Both sheath and watery saliva possessed amylase activities, but polyphenol oxidase and glucose oxidase activities were not detected in either saliva. Peroxidase activity was only detected in salivary sheaths, but only when stink bugs fed on tomato. Proteomic analysis indicated that the peroxidase was likely of plant origin. We also determined that sheath saliva, but not watery saliva elicited the jasmonate inducible defense gene *proteinase inhibitor 2* (*Pin2*), but this induction was only observed when sheaths had been collected from tomato. This indicates that the eliciting factor of the saliva is likely of plant origin. Lastly, neither watery or sheath saliva affected the expression of the salicylate inducible gene pathogenesis related gene (*Pr1a-P4*).

## Introduction

Pentatomid stink bugs include many species that are important pests of crops where they cause feeding damage, especially on seeds and immature fruiting structures. The resulting injury can cause cosmetic damage rendering crops unmarketable or may cause further damage that alters plant maturity, thus inferring with the timing of harvest. Soybean production, in particular, is threatened by a complex of stink bugs that occur throughout most of the soybean production area in the U.S. In addition to many of the common, endemic species such as southern green stink bug (*Nezara viridula*), green stink bug (*Chinavia hilaris or Acrosternum hilare*), and brown stink bug (*Euschistus servus*), a recent complex of stink bugs and associated species including brown marmorated stink bug (*Halyomorpha halys)* (BMSB), the red-banded stink bug (*Piezodorus guildinii*), and the kudzu bug (Plataspidae) (*Megacopta cribraria*) have emerged as new and serious pests in U.S. crops [1]. Although most stink bug species have a wide host range, soybean is often a preferred host for many species and frequently serves as a sink for the buildup of pest populations [2].

In general, stink bugs feed by inserting their needlelike mouthparts or stylets into stems, leaves, blooms, and fruit or seeds. Typically stink bugs either use a lacerate and flush feeding mode or show a preference to feed on leaf vascular tissue which causes minimal mechanical damage [3], [4]. Stink bugs may inject toxic saliva into plant tissues that causes further tissue damage, discoloration or may even cause fruiting structures to abort [5], [6], [7]. Besides causing direct tissue damage, it has been suggested to also carry yeasts that grow within the seeds, although it is unclear if saliva retains yeasts [8]. Feeding damage can delay plant maturity resulting in the abnormal production of new leaflet and pods culminating in the “green bean effect”. Although stink bug saliva is most often implicated as the causal agent in plant damage and the delay in crop maturity, the components of saliva responsible for mediating these adverse effects on crop production are unknown [9]. The damage caused by various species may differ and the differences have been suggested to be due to the components of saliva [6].

The defensive responses of plants to stink bug feeding and saliva are not well known. In general plants respond to chewing herbivores through the jasmonic acid (JA) signaling pathway [10]. The response to sucking insects may be quite different and frequently involves JA-independent signaling mediated by salicylic acid [10], [11]. In a study of two stink bug species with different feeding styles (lacerate and flush vs. vascular feeding), the feeding by the lacerate and flush species (i.e., *Murgantia histrionica*) induced volatile emission consistent with a chewing herbivore, whereas the vascular feeding species (i.e., *Nezara viridula*) as predicted, did not induce volatiles [3]. Although the brown marmorated stink bug has been reported to feed on tree phloem [12], it is unclear if they feed on the vascular tissues of herbaceous plants.

The saliva of hemipterans has been shown in some cases to suppress plant defenses. One of the most classic and elegantly described examples is the suppression of sieve tube plugging by aphid saliva [13]. Other effectors in saliva have been identified that are important in facilitating feeding/and or suppression of defenses such as the proteins MpC002 and MP10 from green peach aphids [14], [15]. Saliva from aphids may also trigger plant systemic responses due to cell wall digestion by gel saliva enzymes that release (e.g., oligogalacturonides) [16]. In the case of the green peach aphid, salivary components also induce defenses responses in Arabidopsis that are independent of the known salicylic acid, jasmonic acid or ethylene signaling pathways [17]. How the saliva of stink bugs may mediate induced defenses is largely unknown.

Very little is known about the saliva of the invasive brown marmorated stink bug (BMSB). The stink bug is a native of Asia, was accidentally introduced in eastern Pennsylvania in the 1990s, and is now rapidly expanding its geographical range across the United States [18]. BMSB is highly polyphagous (>300 hosts) and has become an important pest on pea, soybean, sweet corn, tomato, peppers, eggplant, okra, and many fruits including apple, peach, and cherry, among others [18], [19], [20], [21], [22]. In the northeastern United States, BMSB has emerged as the predominant stink bug species on many cultivated crops and ornamentals [20]. BMSB is also a recent invasive species in Europe with an expanding host range [23]. Because the saliva is believed to be responsible for the major cosmetic and physiological symptoms associated with BMSB feeding, it is important to characterize the salivary components of this insect.

In general, there are two types of saliva produced by most phytophagous Hemiptera such as stink bugs. First, the ***watery saliva*** is involved with digestion of plant food and contains digestive enzymes among other protein components [24]. Watery saliva is thought to be produced by the accessory salivary glands [25]. Second, “gel” saliva is the basis for the formation of the ***salivary sheath*** **** [**26**] **.** The salivary sheath forms a hardened lining around the feeding stylets and the plant tissues [27], [28]. The sheath is necessary to prevent loss of plant juices during feeding by allowing the insect to form a seal around thestylets and the plant tissue. The sheath saliva is released through the salivary canal and rapidly hardens once it is secreted. The sheath adheres to plant surfaces but not to the surface of the stylets. When the insect is finished with a feeding bout, the sheath remains in the plant tissues when the insect withdraws its feeding stylets [29]. A new sheath is formed during each successive feeding bout [27], [30]. Although the salivary sheaths of piercing-sucking insects have been studied for over 60 years, there is still limited progress on the identification of the salivary components responsible for the formation and hardening or gelling of the sheath [26], [31], [32]. The salivary sheath is believed to be a product of the principal salivary glands, whereas the watery saliva is a product of the accessory salivary glands [32], [33].

In this study, we have developed collection methods for both the watery and sheath saliva. With these methods, we can perform enzymatic analysis to identify salivary enzymes, confirm which components of the salivary glands contribute to sheath and watery saliva, and collect sufficient saliva to identify salivary proteins by LC-MS-MS proteomic analysis. Furthermore these tools will be useful for future studies in identifying the specific components of saliva in mediating plant damage and in affecting defenses of their host plants. These methods should be generally applicable for any species of stink bug. In this paper, we describe the salivary glands of BMSB, report on the identification of watery saliva and sheath saliva proteins for BMSB, and determine if BMSB feeding and saliva elicit plant defensive responses in tomato.

## Materials and Methods

### BMSB colony maintenance

BMSB adults were collected from homes and fields in central Pennsylvania and maintained in the laboratory. No specific permissions were required for the collection of the stink bugs because the collections occurred on the private properties of the authors and on the Pennsylvania State University campus at University Park, PA. The collection of these insects did not involve endangered or protected species. BMSB were kept in a growth chamber at 24 C with a 16:8 light dark cycle. Insects were provided with water, organic carrots, grape tomatoes and green beans purchased from the grocery store as well as green bean seedlings (cv. Contender, Burpee, Warminster, PA). Adults typically laid eggs on the underside of the bean leaves and nymphs were reared in the same manner. To determine salivary gland morphology, adult BMSB were dissected and removed glands were placed in Insect Ringer’s solution for photography.

### Watery saliva and sheath collection

To collect watery saliva from BMSB, adult insects were chilled on ice for about five min, then placed ventral side up and observed with a dissecting microscope. As the bugs returned to room temperature, the watery saliva was secreted from the tip of the beak. This saliva was collected into a gel loading pipet tip containing 3 µl of buffer or glycerol. After collection, the buffer and saliva were expelled into a 1.5 ml tube and stored at −80°C. For all enzyme assays and induction experiments, watery saliva was resuspended in pH 7.0 PBS buffer and protein concentrations were determined from Abs at 280 nm measured on a NanoDrop 2000 (Thermo Scientific, Wilmington, DE) and compared to a standard curve of bovine serum albumin.

To collect salivary sheaths, organic grape tomatoes were placed in the BMSB colony cages. After two days, tomatoes were removed and observed with a dissecting microscope. Sheaths were easily identified and carefully removed with forceps to avoid obtaining any tomato tissue, placed in a 1.5 ml tube and stored at −80°C. To collect sheaths from plastic cups, BMSB adults were placed individually in 1 oz portion cups with lids overnight in the growth chamber. After 24 h, bugs were returned to the colony and cups were examined. Salivary sheaths were firmly attached to the plastic and appeared identical to salivary sheaths deposited on plants. The sheaths were carefully removed with forceps, placed into 1.5 ml tubes, and stored at −80°C. For subsequent experiments, sheaths were stored in PBS at 4°C for 4 h, to allow proteins to solubilize. Sheaths in PBS were then centrifuged and the supernatant, containing soluble proteins was collected and protein concentration determined as for watery saliva.

### Polyacrylamide gel electrophoresis (PAGE)

To analyze watery saliva and the content of salivary sheaths by gel electrophoresis, SDS sample buffer was added directly to saliva collected in glycerol or sheaths. Samples were boiled for 5 min then loaded onto a 12% resolving gel and electrophoresed at 200 V. Protein bands were visualized by silver staining [34]. For size determination a protein ladder (Fermentas, Glen Burnie, MD) was also run.

For native PAGE, samples were combined with native sample buffer (0.08 M Tris-HCl, pH 6.8, 30% glycerol and 0.02% bromphenol blue) and loaded onto an 8% acrylamide gel in 1.5 M Tris-HCl, pH 8.8 and electrophoresed at 150 V for 3 h. The gel was then transferred to a peroxidase staining solution (2 mM dianisidine in 0.08 M phosphate buffer pH 7.0, 2% ethanol, 0.15% hydrogen peroxide) to visualize peroxidase activity.

### Proteomics

For proteomic identification of salivary proteins, watery saliva from 110 adult BMSB was collected in 5 mM EDTA in 50 mM Tris-HCl pH 8.0 and stored at −80°C. Salivary sheaths deposited by adult BMSB were collected from tomatoes and stored at – 80°C. For proteomic analysis, sheaths were combined with 5 mM EDTA and 1 M urea in 50 mM Tris-HCl pH 8.0 and allowed to solubilize at 4°C for 2 h then centrifuged to remove insoluble proteins. NanoLC was carried out as previously described [35]. Briefly, proteins were digested with trypsin. Then separated in a Dionex Ultimate 3000 (Milford, MA) and analyzed on an Applied Biosystems Proteomics Analyzer. Peptides were searched against insect, bacteria, and yeast databases (http://blast.ncbi.nlm.nih.gov insects (taxid:6960); bacteria (taxid:2); and yeast (taxid:4932)). Proteins with Total Ion C.I.% greater than 95 were considered high confidence matches.

### Enzyme activity

All chemicals were purchased from Sigma Chemical Co, (St. Louis, MO). Spectrophotometric readings were taken on a Spectramax 190 (Molecular Devices, Silicon Valley, CA). Amylase activity was measured as previously described [36]. Samples were combined with 50 µl 1% starch and incubated at 25°C for 3 min. 100 µl of color reagent (0.01 g/ml 3,5,-dinitrosalicylic acid, 0.3 g/m sodium potassium tartrate tetrahydrate in 0.4 N sodium hydroxide) was added and the mixture was placed in a boiling water bath for 5 min. 1 ml of water was then added and absorbance measured at 540 nm and compared to a maltose standard curve.

To measure peroxidase activity, samples were combined with 3 mM guaiacol, 0.15 % hydrogen peroxide in 0.1 M potassium phosphate buffer pH 7.0. Change in absorbance was measured at 450 nm. Polyphenol oxidase activity was measured by combining samples with 3 mM caffeic acid in 0.1 M potassium phosphate buffer pH 7.0 and measuring the change in absorbance at 450 nm [37]. Glucose oxidase activity was assayed as previously described [38].

### Induction of tomato defenses

To determine if BMSB secretions induced defense genes (*Pin2, Pr1a(P4*) in tomato leaves, the youngest fully expanded leaf of a 4 node tomato plant (cv. Better Boy) was wounded by punching a 4 mm diameter hole and immediately applying 10 μg of watery saliva or salivary sheath in 20 µl PBS. Unwounded plants were used as a negative control and wounded with PBS only was used as a positive wound control. Plants remained in the greenhouse, under super spectrum lights for 24 h, and then 100 mg of tissue was harvested from wounded leaves. RNA extraction and quantitative real time PCR (q-RT-PCR) was performed as described previously [39]. Briefly, tissue was homogenized in liquid nitrogen and RNA extracted using Trizol (Life Technologies). RNA was quantified using a NanoDrop 2000 and 1 µg reverse transcribed with High Capacity Reverse Transcription kit (Applied Biosystems). All q-RT-PCR reactions used FastStart Universal SYBR Green Master Mix (Roche) and were carried out in a 7500 Fast Real –time PCR system (Applied Biosystems). Ubiquitin was used as the endogenous control gene and relative expression was calculated with the median untreated plant as the calibrator. In q-RT-PCR experiments to quantify *Pr1a(P4)*, primers were designed to pathogenesis-related protein 1 (NCBI accession # AJ011520; forward TGTCTCATGGTATTAGCCATATTTCAC; reverse CGTTGTGAACCGCAAGATAGTC). Primers for *Pin2* and ubiquitin were as previously described (39).

We then further determined the role of feeding and saliva on induced defenses by removing the BMSB stylets. To remove the BMSB stylets, adult BMSB were placed on ice for 5 minutes, then with the aid of a dissection microscope, an insect pin was inserted between the stylet and the beak at the beak joint closest to the head. The pin was used to gently lift and remove the stylet from the beak and the stylet was cut off with scissors. Intact BMSB and those with stylets removed were then caged individually on the youngest fully expanded leaf for 24 h. For q-RT-PCR, the area within the cage was harvested and analyzed as described above. This experiment was conducted to separate the effects of BMSB walking and contact on the leaf surface from the effects of feeding and salivation.

## Results

### Morphology

**Figure 1:**
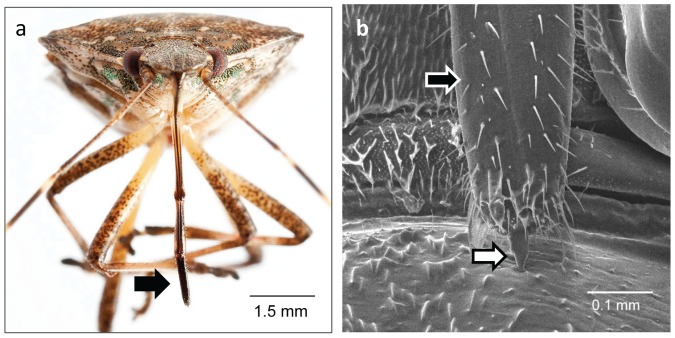
Mouthparts of the brown marmorated stink bug. Photograph (a) and Scanning electron micrograph (b) of BMSB mouthparts. Beak is indicated by black arrow. Stylet extending out of beak is indicated by white arrow.

**Figure 2:**
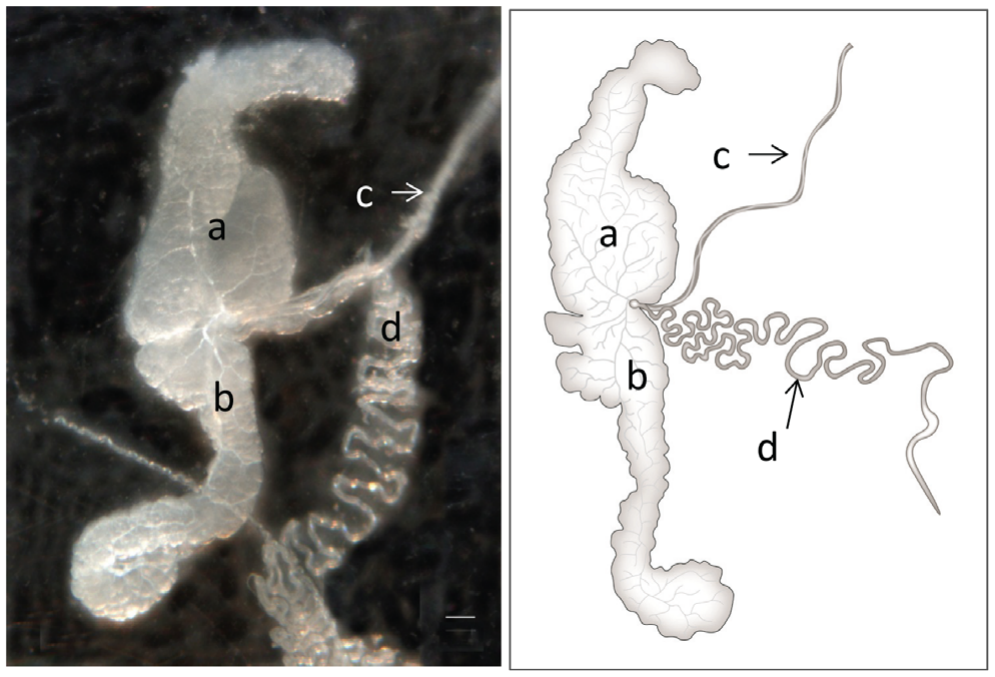
Salivary glands of brown marmorated stink bug. Structure of BMSB salivary glands. (a) anterior lobe of principal gland; (b) posterior lobe of principal gland; (c) salivary duct; (d) accessory gland. Illustration by Nick Sloff.

**Figure 3:**
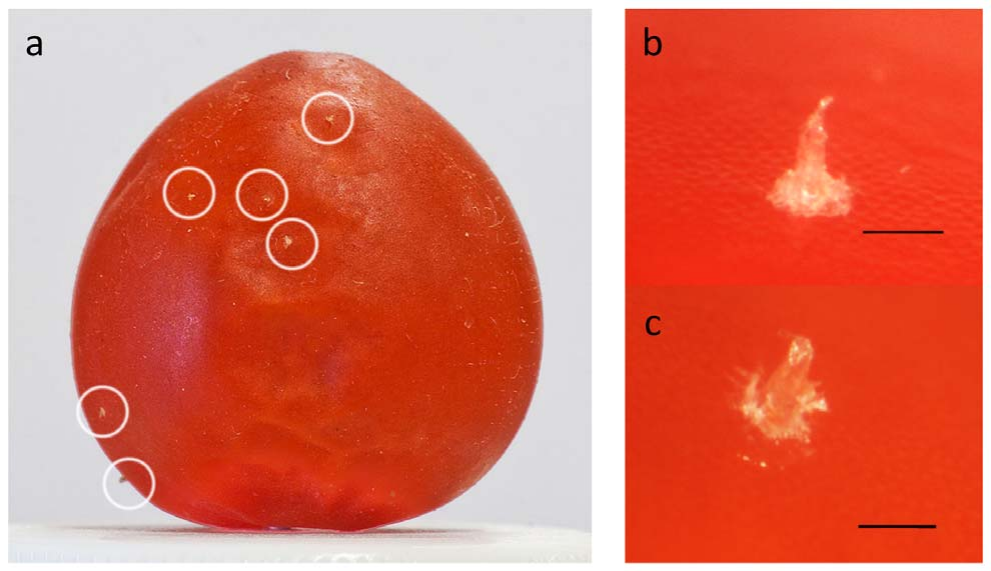
Salivary sheaths of brown marmorated stink bug on tomato. Salivary sheaths on grape tomatoes, bar  = 0.2 mm.

BMSB mouthparts consist of a long beak (Fig. 1a), which houses the stylets inside (Fig. 1b). During feeding, the stylets extend out of the beak and penetrate the plant tissue. The salivary glands are made up of two pairs of glands: principal and accessory (Fig. 2). Salivary glands are located in the thorax adjacent to the gut. The principal gland is made up of 2 lobes, anterior and posterior, with a constriction between the two. At this constriction, the accessory gland, a long thin gland, attaches. Also attached at the constriction is the salivary duct, which connects the glands to the stylets. BMSB produce 2 distinct types of saliva: watery saliva and gel saliva that forms a salivary sheath (Fig. 3). The salivary sheath becomes visible after feeding and may remain attached to the plant at the feeding site. Alternatively it remains on the stylet when feeding ceases and then is expelled during grooming and moving (Movie S1).

### Proteins in Watery Saliva and Sheath

**Figure 4:**
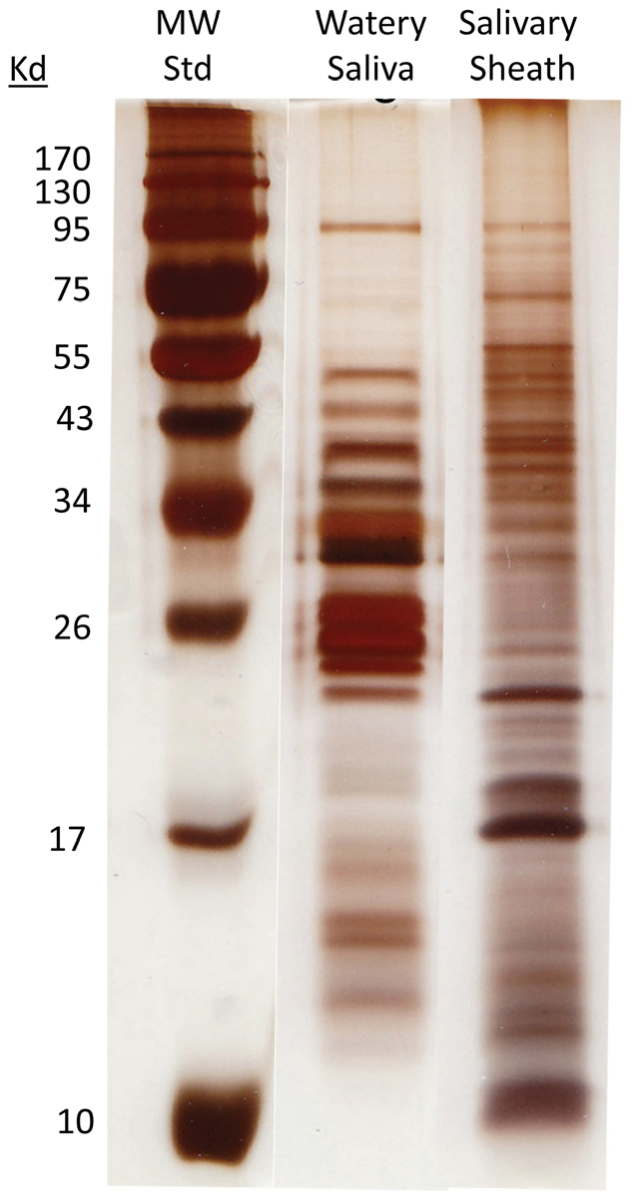
Polyacrylamide gel electrophoresis (PAGE) of BMSB watery saliva and salivary sheaths from BMSB. Watery saliva was collected as described from 6 adult stink bugs. 20 salivary sheaths were collected from tomatoes after stinkbugs had fed on them for 48 hrs. Watery saliva and sheaths were then each combined with 10 µl of SDS sample buffer, boiled for 5 minutes and the entire volume was loaded onto the gel. All three lanes were run on a single gel, the image was edited to remove empty lanes between the samples.

**Figure 5:**
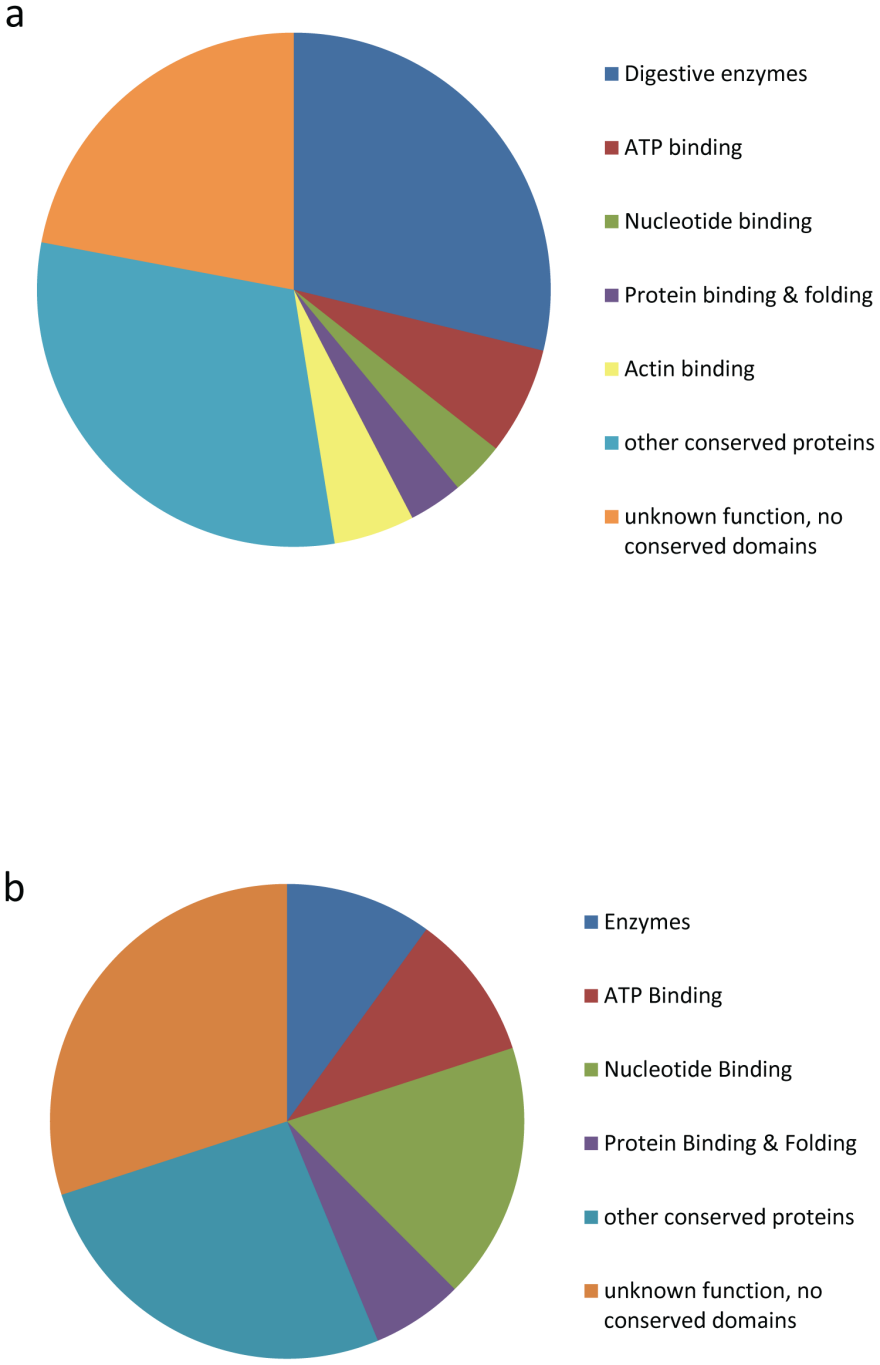
Salivary protein distribution of watery and sheath saliva from the brown marmorated stink bug. Relative abundance of peptides identified by LC- MS/MS from BMSB watery saliva (a) or BMSB salivary sheaths (b).

When watery saliva was run on SDS PAGE next to gut tissue and proteins secreted from the gut, the band patterns were quite distinct (Fig. S1). This provides evidence that the watery saliva we collected does not originate from the gut. SDS PAGE gel electrophoresis also showed that the watery saliva and the salivary sheath have very distinct protein profiles (Fig. 4). Our proteomic data supports this; we did not find any proteins in common between watery saliva and salivary sheath (Tables S1, S2). Watery saliva had 59 peptides with high confidence matches and of these 29% are digestive enzymes (Fig. 5a; Table S1). Other proteins with conserved regions that function in ATP, nucleotide, protein and actin binding were also identified. The soluble fraction of the salivary sheaths contained 80 peptides with high confidence matches (Fig. 5b; Table S2). While only 10% are enzymes, 18% are involved in nucleotide binding. Proteins involved in ATP and protein binding were also identified. Additionally, when peptides were searched against a bacteria or a yeast database, there was no indication that either bacterial or yeast proteins were present in the watery saliva or salivary sheaths.

### Enzymatic survey

**Figure 6:**
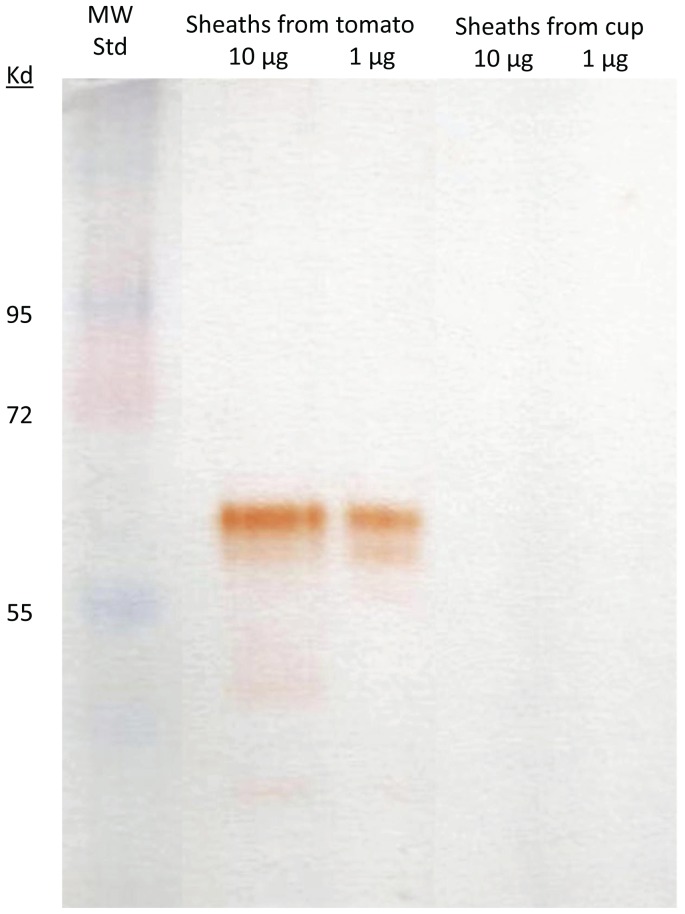
Activity gel stain for peroxidase activity in salivary sheaths of brown marmorated stink bug. Peroxidase stain of native PAGE of BMSB salivary sheaths collected from stink bugs fed grape tomatoes or kept in plastic cups without food.

Both BMSB watery saliva and salivary sheaths contain amylase activity, however sheaths have a higher specific activity than saliva (Table 1). While watery saliva does not have detectable peroxidase activity, significant activity was present in salivary sheaths collected from tomato fed BMSB. We then examined peroxidase activity using native gel electrophoresis to compare the salivary sheaths collected from tomato-fed BMSB and those collected from BMSB held in plastic cups without food. We found that only the sheaths collected from tomato-fed BMSB contained peroxidase activity (Fig. 6). Neither watery saliva nor salivary sheaths contain polyphenol oxidase or glucose oxidase activity.

**Table 1:** Enzyme activities in BMSB watery saliva and salivary sheaths collected from tomatoes.

enzyme	watery saliva	salivary sheath
Amylase (µmole/min/mg protein)	19±0.04	440±176
Peroxidase (ΔOD/min/mg protein)	not detected	902±309
Polyphenol oxidase (ΔOD/min/mg protein)	not detected	not detected
Glucose oxidase (µmole/min/mg protein)	not detected	not detected

Because of the presence of peroxidase activity in sheaths collected from tomato-fed BMSB, we then searched the proteomic sheath data using the *Solanum* database (http://blast.ncbi.nlm.nih.gov Solanum (taxid:4107)) These results were surprising in that a large number of peptides from tomato were successfully identified (N = 116) (Table S3). Included in these data were two peroxidases confirming our observation of peroxidase activity in tomato-fed stink bugs. These results reveal that the protein composition of the sheath is a mixture of insect- and plant-derived proteins.

### Induction of defenses by saliva

**Figure 7:**
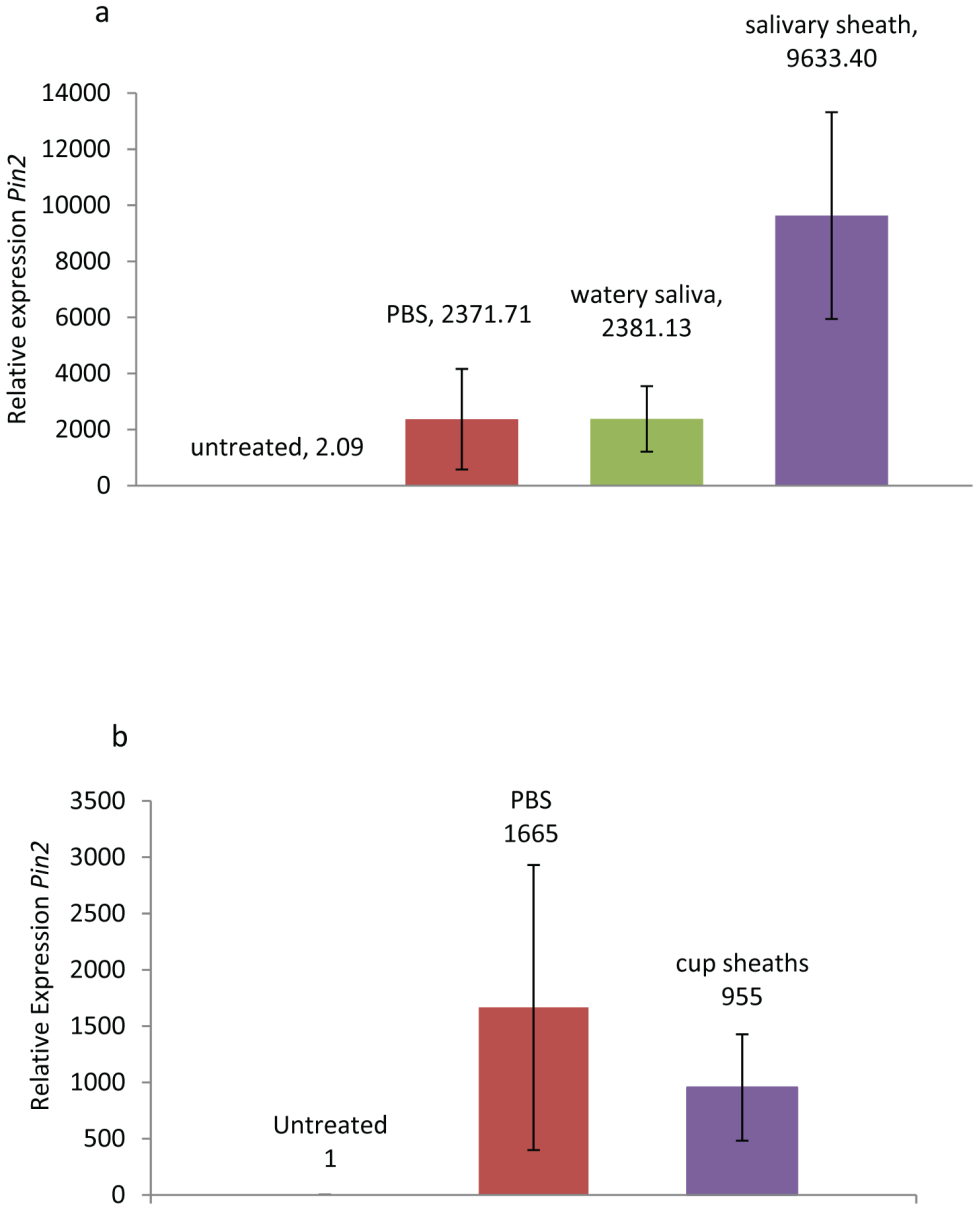
Effect of saliva from brown marmorated stink bug on expression of the proteinase inhibitor 2 (*Pin2*) defense gene in tomato. Relative expression of *Pin2* in tomato leaves 24 h after wounding and application of physiologically buffered saline (PBS), watery saliva or salivary sheath extract from tomatoes (a) or plastic cups (b). For each treatment n = 5 and the median untreated plant was used as the calibrator. (Fisher’s P < 0.05, following ANOVA). Error bars represent +SE.

**Figure 8:**
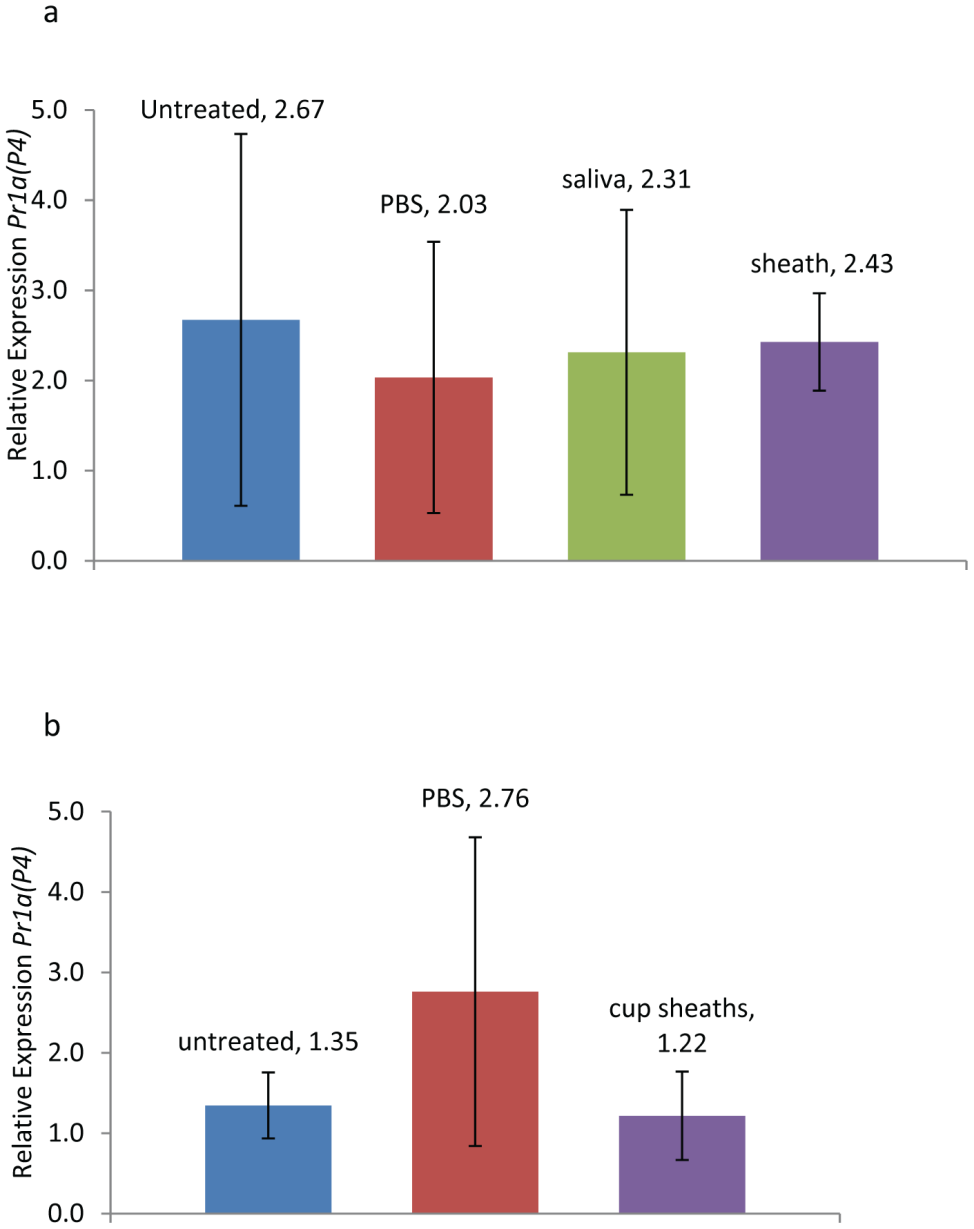
Effect of saliva from brown marmorated stink bug on expression of the pathogenesis related *Pr1a(P4)* defense gene in tomato. Relative expression of *Pr1a(P4)* in tomato leaves 24 h after wounding and application of PBS, watery saliva or salivary sheath extract from tomato-fed BMSB (a) or BMSB held in plastic cups without food (b). For each treatment n = 5 and the median untreated plant was used as the calibrator. (Fisher’s P < 0.05, following ANOVA). Error bars represent +SE.

When watery saliva and salivary sheath extracts were applied to wounded tomato leaves, the leaves with salivary sheath extract had significantly higher *Pin2* expression when compared to plants treated with watery saliva or PBS (Fig. 7A, ANOVA, F = 3.41, p =  0.045). However, when salivary sheaths were collected from plastic cups and applied to wounded tomato leaves, there was no significant difference when compared to PBS treated plants (Fig. 7B, ANOVA, F = 2.69, p = 0.069). When the same plants were analyzed for expression of *Pr1a(P4)*, a gene regulated by salicylic acid, no significant differences were found between any of the treated plants and the untreated control plants (Fig. 8A, ANOVA, F = 0.03 p = 0.992; Fig 8B, ANOVA, F = 0.48 p = 0.630).

**Figure 9:**
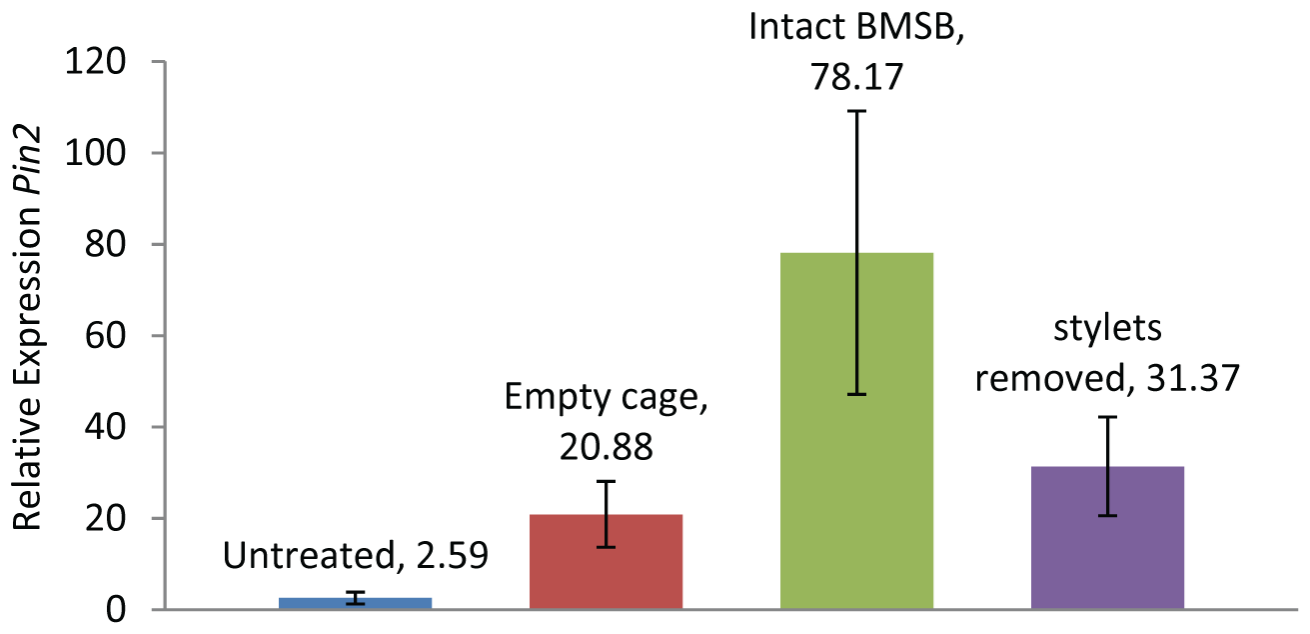
Effect of stylet removal on expression of the proteinase inhibitor 2 (*Pin2*) defense gene in tomato. Relative expression of *Pin2* in tomato leaves 24 h after BMSB were caged on the leaf. For each treatment n = 5 and the median untreated plant was used as the calibrator. (Fisher’s P <.05, following ANOVA). Error bars represent +SE.

To further study the effects of BMSB feeding and saliva on tomato plants, the stylets were removed from the beak and snipped off approximately 2 mm from the head. After this, the bugs continue to live for several days, but they appear unable to feed and cannot produce a salivary sheath. When adult BMSB with intact stylets were caged on tomato leaves, these leaves showed significantly higher expression of *Pin2* than did leaves which had empty cages or cages containing adult BMSB with stylets removed (Fig. 9, ANOVA, F = 3.25, p = 0.033). These results indicate that BMSB feeding and potentially saliva elicits defense gene expression.

## Discussion

The saliva of herbivorous arthropods performs multiple physiological functions including extraoral digestion [40], detoxification [35], [41], [42], [43], evasion of host defenses [44], [45], [46], and protection against microbes [47], [48]. Consistent with a role in digestion, we found several digestive enzymes including amylases, protease, and an esterase in the watery saliva of BMSB (Table S1). We performed an analysis of salivary enzyme activities based upon typical components of saliva (e.g., amylase)[24], salivary enzymes reported to be involved with detoxification of plant secondary compounds (e.g., peroxidase, polyphenol oxidase)[49], [50], [51], [52] and enzymes involved in suppression or induction of plant defenses (e.g., glucose oxidase)[44], [53], [54]. Although glucose oxidase and other GMC-oxidoreductases have been detected in aphids [55], no glucose oxidase activity or GMC-oxidoreductase proteins were detected in BMSB saliva. Gelling and stabilization of the salivary sheath in Hemiptera has been proposed to result from oxidation of low molecular weight compounds by polyphenol oxidase [26], [32] and the absence of any detectable polyphenol oxidase activity in BMSB saliva is noteworthy However, in earlier studies authors used whole salivary glands rather than secreted watery saliva or sheath material. The proteomic analysis of the sheath revealed a phenol oxidase of plant origin (Table S3), but the abundance of this protein was apparently too low to detect enzymatic activity.

Significant peroxidase activity was detected in the salivary sheath (Table 1), suggesting that this enzyme could be involved in sheath formation. However, no peroxidases of insect origin were detected in the watery saliva by proteomic analyses (Table S1). When we examined peroxidase activity via native gel electrophoresis we detected significant activity in sheaths obtained from BMSB feeding on tomato (Fig. 5). However, when BMSB were not provided food, the sheaths deposited on plastic cups did not contain any detectable peroxidase activity (Fig. 5). These results suggested that we should further analyze the sheath proteomic data by searching our results against a *Solanum* database. The results of this analysis were surprising in that we detected a total of 116 *Solanum* proteins, compared to only 46 of insect origin. No evidence for proteins of microbial origin was obtained. Moreover, based upon the total number of peptides identified per protein, the tomato proteins appeared to be major components of the sheath saliva. Extraordinary precaution was exercised to avoid disrupting tomato tissues during the collection of the sheaths. We do not believe that the composition of tomato proteins in the sheath material is a spurious artifact of our collection methods, but represents the natural coalescing of insect and plant derived proteins that occurs during formation of the sheath and subsequent feeding. Accordingly, the presence of peroxidase activity in the sheaths derived from tomato-fed BMSB could be attributed to the presence of two peroxidases detected in the sheath proteome that were *Solanum lycopersicum* proteins (Table S3). Consequently, we were unable to detect any candidate salivary oxidases that are of insect origin and that may function in gel or sheath formation. Nevertheless, it is possible that the oxidases, once polymerized in the sheath, remain insoluble in our extraction protocols and would have escaped detection by our methods.

The salivary protein (ACYPI009881) identified in *Acyrthosiphon pisum* was suggested to be a potential candidate as a structural sheath protein [56]. It has been indicated that this protein shares close similarity to other aphid salivary proteins and thus may represent a highly conserved structural protein among Hemiptera [26]. We blasted our sheath protein sequences against ACYPI009881 and did not find any significant matches.

We found several sheath proteins that are normally associated with microtubule formation and binding. Included in the identifications were two dyneins, which are motor proteins (with ATPase activity) that associate with microtubules [57]. Another identified protein was a lava lamp protein which associates with dyneins [58]. A hyaluronan-mediated motility receptor is another sheath protein that is known to cross-link microtubules, through direct interactions with microtubules and an association with dyneins [59]. A microtubule associated protein xmap215, which enhances microtubule growth rates and remains bound to ends of the microtubules, was also identified [60]. Another protein matched to a putative a nesprin-1; nesprins are high molecular weight actin-binding proteins [61]. The known structural roles of many of these sheath proteins suggest they may contribute to the structure of the salivary sheath. Because sheath formation during feeding is considered an essential part of the feeding process, targeting genes encoding the structural components of the sheath may be an effective strategy using RNA interference.

We did not find any evidence that watery saliva could suppress defense gene expression. Expression of the jasmonate-inducible *Pin2* gene and the salicylate-inducible *Pr1a(P4)* gene was not affected by watery saliva (Figs. 7, 8). In a previous study we found that salivary enzymes with ATPase activity in the caterpillar *Helicoverpa zea* had strong suppressing activity on jasmonate-induced responses [62]. Although multiple proteins present in BMSB watery saliva and sheath possess ATPase activities (e.g., dyneins), their potential effects on induced responses are either masked by other salivary components or their *in vivo* enzymatic activities are insufficient to affect plant responses. In contrast to watery saliva, we found that salivary sheath extracts elicited a significant increase in *Pin 2* expression (Fig. 7a). However, we only observed that sheaths collected from tomato fed BMSB elicited *Pin2*, because sheath extracts collected from BMSB held in plastic cups failed to elicit *Pin2*. Regardless of source, sheath extracts failed to affect *Pr1a(P4)* expression. The observation that sheath extracts from tomato fed BMSB elicit *Pin2* expression indicates that either unique salivary components are produced when BMSB feed on tomato, or that components from tomato present in the sheath are responsible for inducing *Pin2*. It has been noted in aphids that defense responses can also be induced by cell wall degradation products that are produced by cellulase and pectinase, present in the saliva of aphids [63]. It should be noted that we identified a couple of pectinases of tomato origin in the salivary sheaths deposited by BMSB. The specific pectic fragments produced by the action of pectinases are known to elicit defense responses in plants including specifically, *Pin2* in tomato [64].

In summary, we have developed methods for collecting watery and sheath saliva for BMSB that may be utilized for many other stink bug species and perhaps other Hemipterans. Our results indicate that the watery saliva and sheath saliva have very distinct protein profiles. We have made initial findings that sheath saliva may elicit induced plant responses. Future research will include comparative transcriptomics of salivary glands from stink bugs feeding on varied diets. Further characterization of the salivary components that contribute to sheath formation and for eliciting plant responses is now possible.

## Supporting Information

Figure S1 **Silver stained SDS PAGE gel of BMSB watery saliva, gut tissue and secretions from gut tissue.**
(PPTX)

Table S1 **Proteins identified in BMSB watery saliva by Nano LC-MSMS.** Peptides were searched against the NCBI insect database.
(DOCX)

Table S2 **Proteins identified in BMSB salivary sheath by Nano LC-MSMS.** Peptides were searched against the NCBI insect database.
(DOCX)

Table S3 **Proteins identified in BMSB salivary sheath by Nano LC-MSMS.** Peptides were searched against the NCBI Solanum database.
(DOCX)

Movie S1 **Adult BMSB feeding on a tomato fruit.**
(M4V)
