# Supplementary material for: Insights into the Saliva of the Brown Marmorated Stink Bug Halyomorpha halys (Hemiptera: Pentatomidae)
Source: PLoS One. 2014 Feb 26;9(2):e88483. doi: 10.1371/journal.pone.0088483 (PMC3935659; doi:10.1371/journal.pone.0088483)
Supplement: Table S1 — Proteins identified in BMSB watery saliva by Nano LC-MSMS. Peptides were searched against the NCBI insect database. (DOCX) [file pone.0088483.s002.docx]

Table S1: Proteins identified in BMSB watery saliva by Nano LC-MSMS. Peptides were searched against the NCBI insect database

|  | Protein Identification | Organism | NCBI accession # | # of peptides | total ion score | MW | PI |
| --- | --- | --- | --- | --- | --- | --- | --- |
| 1 | serine protease | *Laccotrephes japonensis* | gi\|167614179 | 2 | 201 | 35,336 | 8.5 |
| 2 | alpha-amylase | *Drosophila eugracilis* | gi\|2446949 | 1 | 84 | 29,101 | 5.3 |
| 3 | alpha-amylase | *Apis mellifera mellifera* | gi\|20377081 | 1 | 75 | 55,974 | 7.2 |
| 4 | GA21795 | *Drosophila pseudoobscura pseudoobscura* | gi\|198456735 | 2 | 68 | 284,703 | 5.9 |
| 5 | ACYPI007196 | *Acyrthosiphon pisum* | gi\|239790293 | 1 | 60 | 31,594 | 8.8 |
| 6 | AGAP002715-PA | *Anopheles gambiae str. PEST* | gi\|333468012 | 2 | 57 | 262,571 | 6.4 |
| 7 | hypothetical protein AND_00762 | *Anopheles darlingi* | gi\|312385457 | 2 | 57 | 70,855 | 5.4 |
| 8 | muscle-specific protein 300 | Tribolium castaneum | gi\|270014225 | 3 | 55.0 | 1,760,606 | 5.1 |
| 9 | similar to acyl-CoA delta-9 desaturase | *Nasonia vitripennis* | gi\|156541200 | 2 | 54 | 38,108 | 9.4 |
| 10 | hypothetical protein AND_01746 | *Anopheles darlingi* | gi\|312384701 | 2 | 53 | 144,320 | 8.8 |
| 11 | hypothetical protein AND_07356 | *Anopheles darlingi* | gi\|312380566 | 2 | 53 | 56,876 | 5.5 |
| 12 | GI16332 | *Drosophila mojavensis* | gi\|195133264 | 2 | 53 | 171,963 | 8.3 |
| 13 | GI21311 | *Drosophila mojavensis* | gi\|195124829 | 2 | 52 | 245,435 | 9.1 |
| 14 | hypothetical protein TcasGA2_TC015696 | *Tribolium castaneum* | gi\|270009061 | 3 | 51 | 68,093 | 8.7 |
| 15 | GM18457 | *Drosophila sechellia* | gi\|195342407 | 2 | 51 | 29,518 | 4.8 |
| 16 | GG18471 | *Drosophila erecta* | gi\|194889032 | 1 | 50 | 50,619 | 9.3 |
| 17 | hypothetical protein TcasGA2_TC009918 | *Tribolium castaneum* | gi\|270010512 | 2 | 50 | 144,913 | 6.0 |
| 18 | hypothetical protein SINV_04218 | *Solenopsis invicta* | gi\|322789721 | 3 | 49 | 260,554 | 5.9 |
| 19 | GF12079 | *Drosophila ananassae* | gi\|194754158 | 3 | 48 | 210,952 | 6.4 |
| 20 | hypothetical protein AaeL_AAEL007148 | *Aedes aegypti* | gi\|157115239 | 1 | 48 | 32,429 | 5.1 |
| 21 | GF14625 | *Drosophila ananassae* | gi\|194759646 | 2 | 47 | 102,445 | 9.1 |
| 22 | hypothetical protein AND_13375 | *Anopheles darlingi* | gi\|312375940 | 2 | 47 | 24,376 | 9.0 |
| 23 | hypothetical protein G5I_08466 | *Acromyrmex echinatior* | gi\|332022886 | 2 | 47 | 129,213 | 8.4 |
| 24 | vacuolar protein sorting, putative | *Pediculus humanus corporis* | gi\|242011022 | 2 | 46 | 52,285 | 8.2 |
| 25 | hypothetical protein AND_00750 | *Anopheles darlingi* | gi\|312385465 | 2 | 46 | 118,824 | 8.8 |
| 26 | Esterase FE4 precursor, putative | *Pediculus humanus corporis* | gi\|242007465 | 1 | 46 | 62,709 | 5.6 |
| 27 | esterase 2 | *Liposcelis bostrychophila* | gi\|206730757 | 1 | 46 | 69,201 | 5.1 |
| 28 | GI20027 | *Drosophila mojavensis* | gi\|195120201 | 1 | 45 | 57,212 | 8.7 |
| 29 | hypothetical protein AaeL_AAEL012236 | *Aedes aegypti* | gi\|157131858 | 1 | 45 | 116,369 | 9.9 |
| 30 | hypothetical protein SINV_07132 | *Solenopsis invicta* | gi\|322794893 | 2 | 44 | 178,770 | 10.3 |
| 31 | hypothetical protein TcasGA2_TC010801 | *Tribolium castaneum* | gi\|270014818 | 2 | 43 | 433,746 | 6.7 |
| 32 | GK21304 | *Drosophila willistoni* | gi\|195450320 | 1 | 43 | 27,715 | 6.3 |
| 33 | WD and tetratricopeptide repeats protein 1 | *Culex quinquefasciatus* | gi\|170053319 | 1 | 43 | 76,591 | 5.0 |
