# Supplementary material for: Insights into the Saliva of the Brown Marmorated Stink Bug Halyomorpha halys (Hemiptera: Pentatomidae)
Source: PLoS One. 2014 Feb 26;9(2):e88483. doi: 10.1371/journal.pone.0088483 (PMC3935659; doi:10.1371/journal.pone.0088483)
Supplement: Table S2 — Proteins identified in BMSB salivary sheath by Nano LC-MSMS. Peptides were searched against the NCBI insect database. (DOCX) [file pone.0088483.s003.docx]

Table S2: Proteins identified in BMSB salivary sheath by Nano LC-MSMS. Peptides were searched against the NCBI insect database

|  | **Protein Identification** | ***Organism*** | **NCBI accession #** | **# of peptides** | **total ion score** | **MW** | **PI** |
| --- | --- | --- | --- | --- | --- | --- | --- |
| 1 | hypothetical protein KGM_19474 | *Danaus plexippus* | gi\|357609286 | 2 | 62 | 53181 | 9.3 |
| 2 | hypothetical protein AND_19758 | *Anopheles darlingi* | gi\|312372742 | 3 | 59 | 234442 | 7.9 |
| 3 | dynein-1-beta heavy chain | *Culex quinquefasciatus* | gi\|170052297 | 3 | 59 | 498320 | 6.1 |
| 4 | hypothetical protein SINV_02999 | *Solenopsis invicta* | gi\|322786967 | 3 | 59 | 140655 | 8.8 |
| 5 | PREDICTED: hypothetical protein LOC100164870 | *Acyrthosiphon pisum* | gi\|328724512 | 2 | 56 | 469226 | 5.9 |
| 6 | putative CD98hc amino acid transporter protein | *Danaus plexippus* | gi\|357604365 | 1 | 55 | 65348 | 5.7 |
| 7 | PREDICTED: partitioning defective 3 homolog isoform 1 | *Acyrthosiphon pisum* | gi\|328719478 | 2 | 53 | 161104 | 9.0 |
| 8 | hypothetical protein TcasGA2_TC001594 | *Tribolium castaneum* | gi\|270017242 | 2 | 52.0 | 100964 | 4.7 |
| 9 | GI16715 | *Drosophila mojavensis* | gi\|195135141 | 2 | 51 | 123192 | 6.3 |
| 10 | GF21364 | *Drosophila ananassae* | gi\|194763923 | 3 | 51 | 116064 | 7.3 |
| 11 | hypothetical protein TcasGA2_TC001805 | *Tribolium castaneum* | gi\|270015270 | 2 | 50 | 102006 | 9.4 |
| 12 | PREDICTED: hypothetical protein LOC100677997 | *Nasonia vitripennis* | gi\|345498199 | 2 | 50 | 85295 | 9.5 |
| 13 | kl-3 gamma dynein heavy chain | *Drosophila grimshawi* | gi\|289567851 | 2 | 50 | 530503 | 6.2 |
| 14 | Ras suppressor protein, putative | *Pediculus humanus corporis* | gi\|242012419 | 1 | 49 | 39305 | 6.9 |
| 15 | GF17067 | *Drosophila ananassae]* | gi\|194742554 | 2 | 49 | 59474 | 6.6 |
| 16 | GI13065 | *Drosophila mojavensis* | gi\|195126407 | 2 | 48 | 209383 | 6.3 |
| 17 | PREDICTED: similar to microtubule associated protein xmap215 | *Tribolium castaneum* | gi\|189234292 | 2 | 48 | 213832 | 7.6 |
| 18 | Nesprin-1, putative | *Pediculus humanus corporis* | gi\|242010332 | 2 | 48 | 977831 | 5.8 |
| 19 | PREDICTED: zinc finger protein 729-like | *Apis mellifera* | gi\|110764720 | 2 | 47 | 173510 | 9.0 |
| 20 | hypothetical protein EAG_09928 | *Camponotus floridanus* | gi\|307184709 | 2 | 46 | 22138 | 9.8 |
| 21 | lava lamp protein | *Culex quinquefasciatus* | gi\|269997171 | 2 | 46 | 327059 | 4.8 |
| 22 | GH23978 | *Drosophila grimshawi* | gi\|195064126 | 3 | 36 | 212984 | 7.3 |
| 23 | GA13024 | *Drosophila pseudoobscura pseudoobscura* | gi\|198462127 | 2 | 46 | 332675 | 6.0 |
| 24 | GI12445 | *Drosophila mojavensis* | gi\|195125716 | 1 | 46 | 64038 | 6.2 |
| 25 | hypothetical protein EAG_15211 | *Camponotus floridanus* | gi\|307171301 | 2 | 45 | 77954 | 10.4 |
| 26 | chymotrypsin, putative | *Aedes aegypti* | gi\|157110725 | 2 | 45 | 40300 | 5.5 |
| 27 | hypothetical protein KGM_10255 | *Danaus plexippus* | gi\|357621535 | 2 | 45 | 60190 | 8.9 |
| 28 | GK12921 | *Drosophila willistoni* | gi\|195453629 | 1 | 44 | 48900 | 6.6 |
| 29 | 60S ribosomal protein L4 | *Harpegnathos saltator* | gi\|307205264 | 2 | 44 | 47736 | 11.2 |
| 30 | GK24224 | *Drosophila willistoni* | gi\|195438268 | 1 | 44 | 164434 | 7.7 |
| 31 | conserved hypothetical protein | *Pediculus humanus corporis* | gi\|242021193 | 1 | 44 | 83670 | 5.4 |
| 32 | hypothetical protein TcasGA2_TC001924 | *Tribolium castaneum* | gi\|270012915 | 1 | 44 | 15958 | 6.7 |
| 33 | GH15743 | *Drosophila grimshawi* | gi\|195011463 | 2 | 44 | 254089 | 9.3 |
| 34 | Sugar transporter ERD6-like 6 | *Acromyrmex echinatior* | gi\|332025735 | 1 | 43 | 49140 | 8.9 |
| 35 | PREDICTED: hypothetical protein LOC100575046 | *Acyrthosiphon pisum* | gi\|328705415 | 1 | 43 | 4710 | 5.8 |
| 36 | hypothetical protein SINV_16496 | *Solenopsis invicta* | gi\|322796644 | 1 | 43 | 17468 | 7 |
| 37 | hypothetical protein KGM_09934 | *Danaus plexippus* | gi\|357602156 | 1 | 43 | 76382 | 9.1 |
| 38 | GJ11159 | *Drosophila virilis* | gi\|195385900 | 1 | 43 | 866060 | 4.8 |
| 39 | GA28114 | *Drosophila pseudoobscura pseudoobscura* | gi\|198459812 | 2 | 43 | 115989 | 9.4 |
| 40 | hypothetical protein SINV_02230 | *Solenopsis invicta* | gi\|322792781 | 1 | 43 | 28433 | 8.1 |
| 41 | hypothetical protein G5I_06836 | *Acromyrmex echinatior* | gi\|332024449 | 1 | 43 | 24131 | 5.7 |
| 42 | Hyaluronan mediated motility receptor | *Camponotus floridanus* | gi\|307168587 | 1 | 43 | 117731 | 5.5 |
| 43 | hypothetical protein AND_02671 | *Anopheles darlingi* | gi\|312384035 | 3 | 43 | 459238 | 5.3 |
| 44 | J domain-containing protein | *Culex quinquefasciatus* | gi\|170064958 | 1 | 43 | 13,959 | 9.1 |
| 45 | GF17829 | *Drosophila ananassae* | gi\|194742138 | 1 | 43 | 42262 | 4.8 |
| 46 | putative AlkB, alkylation repair-like protein 2 | *Danaus plexippus* | gi\|357608389 | 1 | 43 | 65841 | 9.4 |
