# Supplementary material for: Insights into the Saliva of the Brown Marmorated Stink Bug Halyomorpha halys (Hemiptera: Pentatomidae)
Source: PLoS One. 2014 Feb 26;9(2):e88483. doi: 10.1371/journal.pone.0088483 (PMC3935659; doi:10.1371/journal.pone.0088483)
Supplement: Table S3 — Proteins identified in BMSB salivary sheath by Nano LC-MSMS. Peptides were searched against the NCBI Solanum database. (DOCX) [file pone.0088483.s004.docx]

Table S3: Proteins identified in BMSB salivary sheath by Nano LC-MSMS. Peptides were searched against the NCBI Solanum database

|  | Protein Identification | *Organism* | NCBI accession # | # of peptides | total ion score | MW | PI |
| --- | --- | --- | --- | --- | --- | --- | --- |
| 1 | glucan endo-1,3-beta-D-glucosidase precursor | *Solanum lycopersicum* | gi\|350534708 | 12 | 1240 | 37,843.3 | 5.4 |
| 2 | Chain A, Crystal Structure Of The Complex Between Pectin Methylesterase And Its Inhibitor Protein | *Solanum lycopersicum* | gi\|62738428 | 13 | 1016 | 34,532.3 | 8.2 |
| 3 | alpha-galactosidase precursor | *Solanum lycopersicum* | gi\|350536051 | 7 | 968 | 44,913.5 | 5.3 |
| 4 | RecName: Full=Acid beta-fructofuranosidase; AltName: Full=Acid invertase; Short=AI; AltName: Full=Acid sucrose | *Solanum lycopersicum* | gi\|124701 | 13 | 907 | 70,053.2 | 5.5 |
| 5 | polygalacturonase-2 precursor | *Solanum lycopersicum* | gi\|350537043 | 13 | 900 | 50,020.0 | 6.4 |
| 6 | pathogenesis-related protein P2 precursor | *Solanum lycopersicum* | gi\|350538353 | 4 | 377 | 16,015.7 | 8.5 |
| 7 | basic 30 kDa endochitinase precursor [] | *Solanum lycopersicum* | gi\|350534566 | 5 | 368 | 34,323.1 | 6.2 |
| 8 | pectinesterase (EC 3.1.1.11) precursor (clone PE1) - tomato | *Solanum lycopersicum* | gi\|82097 | 7 | 368 | 42,580.8 | 9.3 |
| 9 | RecName: Full=Pectinesterase 3; Short=PE 3; AltName: Full=Pectin methylesterase 3; Flags: Precursor | *Solanum lycopersicum* | gi\|6093738 | 5 | 327 | 60,210.8 | 8.3 |
| 10 | pectin methylesterase | *Solanum tuberosum* | gi\|1321997 | 4 | 321 | 24,169.2 | 8.8 |
| 11 | unnamed protein product | *Solanum tuberosum* | gi\|21465 | 5 | 294 | 33,581.8 | 6.5 |
| 12 | beta-galactosidase STBG5 | *Solanum lycopersicum* | gi\|332105897 | 9 | 290 | 92,379.6 | 7.0 |
| 13 | RecName: Full=Endochitinase 4; Flags: Precursor | *Solanum tuberosum* | gi\|1705810 | 4 | 277 | 32,296.3 | 8.6 |
| 14 | lipid transfer-like protein | *Solanum lycopersicum var. cerasiforme* | gi\|300827245 | 5 | 276 | 9,374.8 | 9.2 |
| 15 | Chain A, Nmr Solution Structure Of A New Tomato Peptide | *Solanum lycopersicum* | gi\|186972729 | 3 | 233 | 4,373.0 | 4.8 |
| 16 | 29 kDa chitinase-like thermal hysteresis protein | *Solanum dulcamara* | gi\|30526289 | 3 | 219 | 28,826.7 | 8.9 |
| 17 | non-specific lipid-transfer protein 2 precursor | *Solanum lycopersicum* | gi\|350535717 | 4 | 176 | 11,476.6 | 8.0 |
| 18 | putative non-specific lipid transfer protein b1 | *Solanum tuberosum* | gi\|156118344 | 4 | 175 | 11,485.8 | 8.7 |
| 19 | pectin methyl esterase | *Solanum tuberosum* | gi\|319993027 | 2 | 172 | 59,745.5 | 7.1 |
| 20 | osmotin-like protein | *Solanum nigrum* | gi\|56787638 | 3 | 171 | 26,796.4 | 6.5 |
| 21 | fruit-specific protein | *Solanum lycopersicum* | gi\|350536509 | 3 | 162 | 10,691.4 | 7.5 |
| 22 | acidic 27 kDa endochitinase precursor | *Solanum lycopersicum* | gi\|350534512 | 3 | 129 | 26,566.6 | 4.7 |
| 23 | glucan endo-1,3-beta-D-glucosidase precursor | *Solanum lycopersicum* | gi\|350534760 | 1 | 126 | 37,838.8 | 9.7 |
| 24 | RecName: Full=Suberization-associated anionic peroxidase 1; AltName: Full=TMP1; Flags: Precursor |  | gi\|129807 | 5 | 125 | 38,725.1 | 4.9 |
| 25 | non-specific lipid transfer protein precursor | *Solanum lycopersicum* | gi\|350535973 | 2 | 119 | 11,901.9 | 8.7 |
| 26 | RecName: Full=Cysteine proteinase 3; Flags: Precursor | *Solanum lycopersicum* | gi\|2499879 | 4 | 101 | 38,919.7 | 8.6 |
| 27 | non-specific lipid transfer protein | *Solanum sogarandinum* | gi\|110962345 | 2 | 97 | 11,642.7 | 8.4 |
| 28 | putative non-specific lipid transfer protein StnsLTP | *Solanum tuberosum* | gi\|21952514 | 3 | 96 | 11,502.9 | 9.2 |
| 29 | dicyanin precursor | *Solanum lycopersicum* | gi\|350535054 | 1 | 92 | 34,166.1 | 8.9 |
| 30 | ethylene-responsive proteinase inhibitor 1 precursor | *Solanum lycopersicum* | gi\|350538787 | 2 | 85 | 13,151.9 | 5.3 |
| 31 | RecName: Full=Unknown protein 1 | *Solanum lycopersicum* | gi\|223635806 | 1 | 82 | 1,490.7 | 4.4 |
| 32 | enolase | *Solanum lycopersicum* | gi\|350538295 | 3 | 72 | 47,768.4 | 5.7 |
| 33 | Putative gag-pol polyprotein, identical | *Solanum demissum* | gi\|47824970 | 10 | 62 | 172,014.0 | 9.2 |
| 34 | unknown | *Solanum tuberosum* | gi\|77999255 | 3 | 60 | 38,412.8 | 8.5 |
| 35 | osmotin 81 | *Solanum tuberosum* | gi\|30145507 | 2 | 60 | 19,021.9 | 8.6 |
| 36 | polygalacturonase-like protein-like | *Solanum tuberosum* | gi\|81074755 | 2 | 58 | 51,899.0 | 5.7 |
| 37 | putative Pto-like serine/threonine kinase | *Solanum berthaultii* | gi\|15054741 | 3 | 54 | 19,441.9 | 6.1 |
| 38 | defensin protein | *Solanum pimpinellifolium* | gi\|133711825 | 2 | 51 | 8,411.9 | 9.0 |
| 39 | osmotin-like protein | *Solanum tuberosum* | gi\|28864860 | 3 | 49 | 16,589.8 | 8.6 |
| 40 | peroxidase precursor | *Solanum lycopersicum* | gi\|350539341 | 4 | 48 | 34,902.1 | 4.6 |
| 41 | osmotin-like protein | *Solanum tuberosum* | gi\|28864852 | 3 | 47 | 16,465.6 | 8.3 |
| 42 | osmotin-like protein | *Solanum tuberosum* | gi\|28864864 | 2 | 47 | 17,753.1 | 6.4 |
| 43 | CT099 | *Solanum habrochaites* | gi\|61968968 | 2 | 46 | 29,403.4 | 7.9 |
| 44 | osmotin-like protein | *Solanum phureja* | gi\|53830832 | 2 | 46 | 27,314.6 | 6.2 |
| 45 | polyprotein-like | *Solanum chilense* | gi\|13540823 | 7 | 46 | 152,218.5 | 9.0 |
| 46 | invertase | *Solanum lycopersicum* | gi\|350538065 | 2 | 45 | 72,786.6 | 6.2 |
| 47 | beta-galactosidase precursor | *Solanum lycopersicum* | gi\|350539595 | 1 | 44 | 93,275.9 | 6.2 |
| 48 | RNA polymerase IV second largest subunit | *Solanum lycopersicum* | gi\|67515354 | 6 | 44 | 127,457.8 | 8.3 |
| 49 | fructokinase 3 | *Solanum lycopersicum* | gi\|350534424 | 2 | 44 | 41,461.3 | 5.6 |
| 50 | mannan endo-1,4-beta-mannosidase 4 precursor | *Solanum lycopersicum* | gi\|350539319 | 3 | 42 | 45,309.8 | 8.9 |
| 51 | enolase | *Solanum lycopersicum* | gi\|1161573 | 3 | 42 | 35,073.1 | 6.3 |
| 52 | aspartate carbamoyltransferase | *Solanum tuberosum* | gi\|21535795 | 2 | 41 | 42,575.8 | 6.1 |
| 53 | RecName: Full=DNA-directed RNA polymerase subunit beta''; AltName: Full=PEP; AltName: Full=Plastid-encoded RNA polymerase subunit beta''; Short=RNA polymerase subunit beta'' | *Solanum tuberosum* | gi\|90111014 | 7 | 39 | 157,059.0 | 9.3 |
| 54 | ACRE 276-like protein | *Solanum tuberosum* | gi\|118490015 | 3 | 40 | 79,235.4 | 8.6 |
| 55 | xyloglucan-specific fungal endoglucanase inhibitor protein precursor | *Solanum lycopersicum* | gi\|350536487 | 2 | 39 | 46,607.5 | 7.9 |
| 56 | ABA 8'-hydroxylase CYPA3 variant 1 | *Solanum tuberosum* | gi\|328461713 | 3 | 39 | 50,725.4 | 8.6 |
| 57 | putative cullin protein | *Solanum lycopersicum* | gi\|3687389 | 3 | 39 | 71,734.0 | 8.3 |
| 58 | putative disease resistance protein | *Solanum demissum* | gi\|48209881 | 10 | 38 | 297,005.5 | 5.4 |
| 59 | flowering locus T protein | *Solanum tuberosum* | gi\|282153480 | 3 | 38 | 19,737.0 | 7.8 |
| 60 | beta-galactosidase STBG2 | *Solanum lycopersicum* | gi\|332105893 | 4 | 35 | 99,763.4 | 6.2 |
| 61 | RNA polymerase II second largest subunit | *Solanum lycopersicum* | gi\|350539902 | 6 | 35 | 137,157.9 | 7.2 |
| 62 | verticillium wilt disease resistance protein | *Solanum lycopersicum* | gi\|237899595 | 3 | 35 | 117,186.7 | 5.5 |
| 63 | Gag-pol protein, putative | *Solanum demissum* | gi\|113205218 | 8 | 35 | 176,510.6 | 9.0 |
| 64 | SNKR2GH2 protein | *Solanum schenckii* | gi\|302594427 | 4 | 35 | 97,744.8 | 6.9 |
| 65 | 2S seed albumin-1 large subunit | *Solanum lycopersicum* | gi\|119116673 | 1 | 34 | 7,497.6 | 5.2 |
| 66 | RecName: Full=Respiratory burst oxidase homolog protein C; AltName: Full=NADPH oxidase RBOHC; AltName: Full=StRBOHC | *Solanum tuberosum* | gi\|166199749 | 6 | 34 | 105,235.6 | 9.0 |
| 67 | Disease resistance protein, putative | *Solanum demissum* | gi\|113205208 | 8 | 34 | 291,028.7 | 5.4 |
| 68 | sesquiterpene synthase | *Solanum lycopersicum* | gi\|356460901 | 5 | 34 | 64,793.7 | 5.2 |
| 69 | 42KDa chitin-binding protein | *Solanum lycopersicum var. cerasiforme* | gi\|269935957 | 1 | 33 | 32,333.9 | 5.7 |
| 70 | RecName: Full=Disease resistance protein RGA2; AltName: Full=Blight resistance protein RPI; AltName: Full=RGA2-blb | *Solanum bulbocastanum* | gi\|46576968 | 4 | 31 | 110,276.3 | 7.2 |
| 71 | RAV1 | *Solanum lycopersicum* | gi\|164458454 | 4 | 33 | 42,195.4 | 9.2 |
| 72 | late blight resistance protein Rpi-blb2 | *Solanum bulbocastanum* | gi\|74040324 | 4 | 33 | 146,043.3 | 5.0 |
| 73 | RecName: Full=Acidic endochitinase pcht28; Flags: Precursor | *Solanum chilense* | gi\|2493672 | 1 | 33 | 27,551.3 | 6.3 |
| 74 | Polyprotein, putative | *Solanum demissum* | gi\|113205142 | 2 | 33 | 100,059.8 | 8.9 |
| 75 | AML1 | *Solanum tuberosum* | gi\|47834705 | 4 | 33 | 92,238.8 | 6.2 |
| 76 | putative inward rectifying potassium channel | *Solanum tuberosum* | gi\|2225997 | 2 | 32 | 97,039.0 | 6.3 |
| 77 | Gag-pol polyprotein, putative | *Solanum demissum* | gi\|113205301 | 6 | 32 | 92,004.2 | 8.6 |
| 78 | salt responsive protein 2 | *Solanum lycopersicum* | gi\|350536071 | 2 | 32 | 57,130.6 | 6.3 |
| 79 | flavonoid 3',5'-hydroxylase | *Solanum tuberosum* | gi\|56269731 | 4 | 32 | 56,913.4 | 8.5 |
| 80 | DNA (cytosine-5)-methyltransferase | *Solanum lycopersicum* | gi\|350536241 | 7 | 31 | 174,805.6 | 6.0 |
| 81 | TPR domain containing protein, putative | *Solanum demissum* | gi\|113205415 | 3 | 31 | 59,717.5 | 8.0 |
| 82 | propolyphenol oxidase | *Solanum tuberosum* | gi\|404585 | 3 | 31 | 65,700.7 | 6.4 |
| 83 | COSII_At3g16150 | *Solanum peruvianum* | gi\|224980352 | 3 | 30 | 6,680.3 | 4.8 |
| 84 | vicilin | *Solanum lycopersicum* | gi\|166053040 | 3 | 30 | 66,136.9 | 8.2 |
| 85 | Hop-interacting protein THI007 | *Solanum lycopersicum* | gi\|365222860 | 4 | 30 | 66,481.7 | 5.5 |
| 86 | temperature-induced lipocalin | *Solanum tuberosum* | gi\|77744887 | 3 | 30 | 21,441.8 | 5.5 |
| 87 | resistance protein PSH-RGH7 | *Solanum tuberosum* | gi\|164598918 | 4 | 30 | 107,034.0 | 5.4 |
| 88 | 26S proteasome AAA-ATPase subunit RPT4a | *Solanum tuberosum* | gi\|24745880 | 4 | 30 | 44,690.4 | 8.2 |
| 89 | anthocyanin synthase | *Solanum cardiophyllum* | gi\|339740056 | 2 | 30 | 51,095.2 | 5.2 |
| 90 | NBS-LRR resistance protein-like protein | *Solanum lycopersicum* | gi\|115381106 | 4 | 30 | 143,747.0 | 5.1 |
| 91 | RecName: Full=Glucan endo-1,3-beta-glucosidase, basic isoform 1; AltName: Full=(1->3)-beta-glucan endohydrolase; Short=(1->3)-beta-glucanase; AltName: Full=Beta-1,3-endoglucanase; Flags: Precursor | *Solanum tuberosum* | gi\|1706543 | 1 | 29 | 36,998.6 | 6.7 |
| 92 | Hop-interacting protein THI109 | *Solanum lycopersicum* | gi\|365222916 | 4 | 29 | 79,116.3 | 5.4 |
| 93 | hexokinase | *Solanum lycopersicum* | gi\|350534620 | 3 | 29 | 53,709.5 | 6.3 |
| 94 | starch synthase IV | *Solanum lycopersicum* | gi\|350538831 | 1 | 29 | 113,787.6 | 5.6 |
| 95 | sucrose synthase | *Solanum lycopersicum* | gi\|350534492 | 4 | 29 | 92,387.5 | 5.9 |
| 96 | Putative disease resistance protein, identical | *Solanum demissum* | gi\|49533783 | 3 | 28 | 87,444.9 | 8.3 |
| 97 | heat shock protein 70 | *Solanum lycopersicum* | gi\|350537379 | 3 | 28 | 74,243.5 | 5.4 |
| 98 | fumarase | *Solanum tuberosum* | gi\|1488652 | 2 | 28 | 53,348.0 | 6.5 |
| 99 | R1 | *Solanum tuberosum* | gi\|53831187 | 2 | 28 | 59,581.9 | 5.8 |
| 100 | phospholipase PLDa2 | *Solanum lycopersicum* | gi\|350538613 | 3 | 27 | 92,656.8 | 6.3 |
| 101 | CC-NBS-LRR protein | *Solanum tuberosum* | gi\|149786544 | 3 | 27 | 149,737.9 | 6.2 |
| 102 | copia LTR rider | *Solanum lycopersicum]* | gi\|133711804 | 2 | 27 | 148,160.0 | 8.7 |
| 103 | folylpolyglutamate synthase | *Solanum pennellii* | gi\|325516272 | 4 | 27 | 52,016.9 | 6.8 |
| 104 | maturase K | *Solanum trisectum* | gi\|156628854 | 2 | 27 | 36,816.0 | 9.7 |
| 105 | sialyltransferase-like protein | *Solanum lycopersicum]* | gi\|350538381 | 4 | 27 | 53,446.4 | 9.3 |
| 106 | S-RNase | *Solanum neorickii* | gi\|21623713 | 2 | 26 | 24,827.4 | 8.6 |
| 107 | cysteine-rich receptor-like protein kinase | *Solanum nigrum* | gi\|327493191 | 2 | 26 | 19,280.4 | 8.6 |
| 108 | hypothetical protein SDM1_42t00016 | *Solanum demissum* | gi\|113205321 | 2 | 26 | 13,265.4 | 8.7 |
| 109 | Ve resistance gene analog | *Solanum tuberosum* | gi\|16930096 | 3 | 25 | 31,142.7 | 6.0 |
| 110 | NL25 | *Solanum tuberosum* | gi\|3947733 | 3 | 25 | 60,914.9 | 6.5 |
| 111 | plant resistance protein | *Solanum lycopersicum]* | gi\|350538941 | 3 | 25 | 144,264.3 | 5.0 |
| 112 | GPA2-like NBS-LRR protein | *Solanum nigrum* | gi\|16326619 | 4 | 24 | 54,815.3 | 8.4 |
| 113 | Myb-like DNA-binding protein, putative | *Solanum demissum* | gi\|113205212 | 3 | 24 | 58,033.6 | 6.0 |
| 114 | RSI6 | *Solanum tuberosum* | gi\|338815371 | 3 | 24 | 28,902.4 | 6.5 |
| 115 | cyclin A2 | *Solanum lycopersicum]* | gi\|350537079 | 2 | 24 | 53,886.6 | 8.6 |
| 116 | RB | *Solanum chacoense* | gi\|359719422 | 1 | 24 | 19,132.1 | 6.9 |
